# The Trend of Cholecystectomies After the Introduction of Laparoscopic Surgery in a District Hospital in Abuja, North Central Nigeria

**DOI:** 10.7759/cureus.41122

**Published:** 2023-06-29

**Authors:** Michael E Aghahowa, Iliya K Salu, Rosemary M Nwokorie, Oku S Bassey, Sabastine N Esomonu, Mtaku B Gali

**Affiliations:** 1 Surgery, Asokoro District Hospital, Abuja, NGA; 2 Surgery, Nile University of Nigeria, Abuja, NGA; 3 Anesthesiology, Asokoro District Hospital, Abuja, NGA; 4 Anesthesiology, Nile University of Nigeria, Abuja, NGA; 5 Radiology, Asokoro District Hospital, Abuja, NGA; 6 Epidemiology and Biostatistics, Federal Capital Territory Primary Healthcare Board, Abuja, NGA

**Keywords:** trend, introduction, laparoscopy, cholelithiasis, cholecystectomy

## Abstract

Background

Laparoscopic cholecystectomy is not readily available in secondary care hospitals in Nigeria, even though it is now the gold standard for the treatment of cholelithiasis and other gallbladder diseases worldwide. Thus, many hospitals in Nigeria still offer open cholecystectomies. This retrospective study investigated the trend of cholecystectomies performed in the general surgery unit of a district hospital in Abuja before and after the commencement of laparoscopic surgery services in 2016.

Methodology

This retrospective study was conducted in Asokoro District Hospital, Abuja, Nigeria The records of all patients who underwent a cholecystectomy from January 2000 to December 2019 were retrieved and analyzed for the number, types, and rate of cholecystectomies performed per year. All open cholecystectomies were performed via a right subcostal incision, whereas all laparoscopic cholecystectomies were performed via the standard four-port incisions approach.

Results

A total of 96 patients underwent cholecystectomies from January 2000 to December 2019. In total, 50 (52.08%) open cholecystectomies were performed in 20 years with a yearly average of 2.5, and 46 (47.92%) laparoscopic cholecystectomies were performed in four years with a yearly average of 11.5. The trend of open cholecystectomies in four years dropped from three (30%) in 2016 to one (5.26%) in 2019, whereas laparoscopic cholecystectomies increased from seven (70%) to 18 (94.74%) within the same period.

Conclusions

There is a drop in the trend of open cholecystectomies and an increase in both laparoscopic and total cholecystectomies in our hospital. We recommend adequate capacity and subsidized laparoscopic cholecystectomy for secondary healthcare facilities in Nigeria.

## Introduction

Carl Langenbuch performed the first successful open cholecystectomy (OC) in 1882, and for more than 100 years, it has been the standard treatment for symptomatic gallbladder stones [1]. Laparoscopic cholecystectomy (LC), which was first performed by the German surgeon Muehe in 1986, and then in France by Phillip Mouret in 1987, has replaced OC for the elective surgical management of symptomatic gallstones and other gallbladder diseases globally as the gold standard. LC has the advantages of being a minimally invasive procedure with less bleeding, reduced postoperative discomfort and pain, shorter hospital stay (of approximately three days), no disfiguring scar, rapid convalescence, and earlier return to work [1-4]. This revolutionized treatment of gallbladder diseases has not only supplanted OC but has also ended attempts for the non-invasive management of gallstones, such as extracorporeal shock wave and bile salt therapy [1].

OC remains the common surgical treatment for gallstones (cholelithiasis) in most hospitals in Nigeria [5]. LC is now the gold-standard treatment of symptomatic gallstones and other benign diseases of the gallbladder worldwide, and the key, similar to open surgery, is the identification and safe dissection of Calot’s triangle to avoid complications such as injuries to the biliary ducts and cystic artery [3]. Despite the advantages of LC, it is yet to be widely practiced in many developing countries of Africa, and only a few health institutions in Nigeria have the infrastructure and capacity to perform LC [6]. However, LC is currently being performed in some public tertiary healthcare facilities such as teaching hospitals, federal medical centers, and a few private hospitals in Nigeria [6]. The capacity to perform minimally invasive surgical procedures is even more farfetched in most secondary healthcare facilities in a low and middle-income country (LMIC) such as Nigeria. This is probably due to the poor economic standings at the sub-national tiers of government, the dearth of appropriate personnel and other infrastructure, as well as other logistical issues, such as erratic power supply and the non-availability of consumables used for laparoscopic surgery. Available literature does not indicate any secondary healthcare facility or general hospital in Nigeria where LC is routinely undertaken. Therefore, OC is still mostly offered by a majority of these centers in Nigeria [5]. However, the capacity for both OC and LC is available in Asokoro District Hospital, which is a 120-bed, public secondary healthcare facility in Abuja, North Central Nigeria. It is owned by the Federal Capital Territory (FCT) Administration and is located in Asokoro, one of the low-density, urban districts in the Federal Capital City (FCC) of Abuja. Asokoro District Hospital receives patients from within the FCT and six neighboring states, with about 2,000 new patients seen in the general surgery outpatient clinic annually.

Although the first OC in Asokoro District Hospital, Abuja was done in 2000 when it was commissioned, the capacity for laparoscopic surgery was not available until 14 years after in 2014 when the hospital employed the necessary personnel and procured a laparoscopy tower. It is the first known government-owned secondary healthcare facility to acquire the capacity for laparoscopic surgery in Nigeria. Many patients who would have traveled long distances to tertiary healthcare centers in Nigeria or even go overseas now have the opportunity of undergoing LC at a cheaper rate in a district hospital in Abuja, Nigeria.

This study focused on the cholecystectomies performed by the general surgery unit of a general hospital in Abuja before and after the acquisition of a laparoscope as well as the lessons learned. The aim was to determine the trend of cholecystectomies after the introduction of laparoscopic surgery services in Asokoro District Hospital, Abuja, in North Central Nigeria.

## Materials and methods

In this retrospective study, the case notes, electronic medical records, and operation registers of all 50 patients who underwent OC over a period of 20 years, from January 2000 to December 2019, and 46 patients who underwent LC over a period of four years, from January 2016 to December 2019, at the general surgery unit of Asokoro District Hospital were reviewed. The variables analyzed were the number, types, and rate of cholecystectomies performed per year. The diagnosis of benign gallbladder disease was made clinically, with symptoms of pain in the right upper quadrant of the abdomen, a positive pointing sign, and a transabdominal ultrasound scan. Routine complete blood counts and biochemical profiles were done to exclude anemia and identify abnormal renal and liver functions, respectively. Chest radiography was done where necessary.

The choice of open or laparoscopic surgery was entirely that of the patient after informed voluntary consent. Double consent for conversion to OC was taken from all patients who underwent LC. All patients were administered general anesthesia through cuffed endotracheal intubation, and a nasogastric tube passed after the induction of anesthesia was removed within 24 hours postoperatively. OC was done via Kocher’s (right subcostal) incision and a RediVac™ (vacuum) drain left in the gallbladder bed, while LC was done via the standard four-port incisions approach, and no drain was used. All cholecystectomies were done by the same team of one certified laparoscopic surgeon and another general surgeon who were assisted by different medical officers. The postoperative follow-up of patients was for 12 months. The emphasis of this study was on the trend of OCs and LCs after the introduction of laparoscopic surgery services in our healthcare facility. The data were analyzed using SPSS version 23 (IBM Corp., Armonk, NY, USA). Categorical data were expressed as frequencies and percentages. This review was approved by the Ethics and Research Committee of the hospital.

## Results

A total of 96 cholecystectomies were performed over a 20-year period from January 2000 to December 2019 with an average of 4.8 per year. Of these, 50 (52.08%) were OCs over the entire period, with a yearly average of 2.5, while 46 (47.92%) were LCs in the last four of the 20-year period, with a yearly average of 11.5 (Table 1).

**Table 1:** Distribution of years, number, and types of cholecystectomies.

	Types of cholecystectomies (N = 96)		
Year	Open (n = 50), n (%)	Laparoscopic (n = 46), n (%)	Total, n (%)
2000–2004	7 (100)	0 (0)	7 (100)
2005–2009	16 (100)	0 (0)	16 (100)
2010–2014	18 (100)	0 (0)	18 (100)
2015	1 (100)	0 (0)	1 (100)
2016	3 (30)	7 (70)	10 (100)
2017	2 (18.2)	9 (81.8)	11 (100)
2018	2 (14.3)	12 (85.7)	14 (100)
2019	1 (5.3)	18 (94.7)	19 (100)

**Figure 1:**
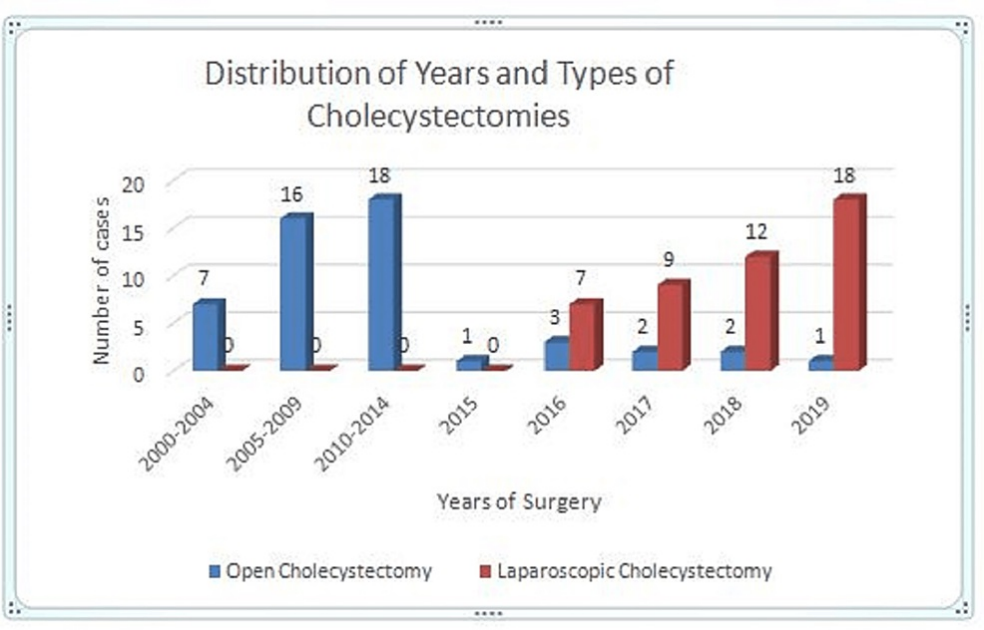
Distribution of the number and types of cholecystectomies according to year.

Further analysis revealed that there were 42 (100%) OCs in 16 years (2000-2015), with an average of 2.6 per year before the deployment of laparoscopy in 2016. However, after the introduction of laparoscopy in 2016, 54 total cholecystectomies were done in four years (2016-2019), with an average of 13.5 per year. Of these, OC accounted for only eight (14.8%) while LC accounted for 46 (85.2%) cases, with an average of two OCs per year and 11.5 LCs per year, respectively. Moreover, out of the total 54 cholecystectomies done in four years, OC accounted for three (5.5%), two (3.7%), two (3.7%), and one (1.9%) cases, in 2016, 2017, 2018, and 2019, respectively, whereas LC accounted for seven (13%), nine (16.7%), 12 (22.2%), and 18 (33.3%) cases, respectively, during the same period (Table 1, Figure 1).

**Figure 2:**
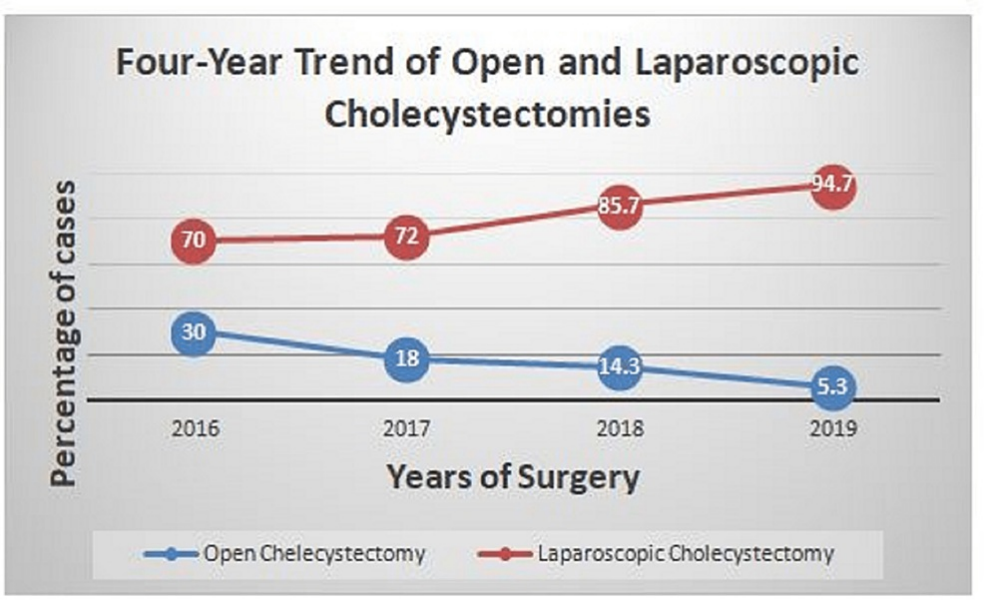
Trend of open and laparoscopic cholecystectomies in percentages.

There was a steep rise in the uptake of LC soon after the acquisition of the laparoscope as evidenced by seven (70%), nine (82%) 12 (85.7%), and 18 (94.7%) out of the yearly total cholecystectomies performed while that of OC dropped yearly from three (30%), two (18%), two (14.3%), to one (5.3%) in 2016, 2017, 2018, and 2019, respectively (Table 2, Figure 2).

**Table 2:** Distribution of open and laparoscopic cholecystectomies over a four-year period.

Year of surgery	Open (n = 8), n (%)	Laparoscopic (n = 46), n (%)	Total
2016	3 (30%)	7 (70%)	10 (100%)
2017	2 (18%)	9 (82%)	11 (100%)
2018	2 (14.3%)	12 (85.7%)	14 (100%)
2019	1 (5.3%)	18 (94.7%)	19 (100%)

## Discussion

The introduction of laparoscopy into the surgical armamentarium of Asokoro District Hospital, Abuja in 2014 revolutionized the trend of cholecystectomies in this secondary care hospital. For two years after the acquisition of the tower, no LC was done because the cost of the procedure precluded some patients from accessing the service. Though the costs of healthcare services are highly subsidized by the FCT Administration, appropriately subsidized unit costing for both LC and OC had to be undertaken to bring the cost of both procedures to near-par to improve access to laparoscopic services. The cost of LC was reduced from N125,000 (equivalent to $250) to N75,000 (equivalent to $150) per patient, while the cost of OC was increased from N50,000 (equivalent to $100) to N70,000 (equivalent to $140) per patient. This price modulation reduced the financial bottlenecks and enabled the first LC to be performed in 2016. Thereafter, the choice of OC dropped remarkably with most patients opting for LC.

From 2000 to 2019, a total of 50 cholecystectomies were done in the 20-year period under review. The number of OC fluctuated between one and four years until it reached its highest peak of seven in 2013, followed by a declining trend to five in 2014, three in 2016, and, finally, one in 2019 (Table 1). The percentage of OC gradually dropped from 5.5% in 2016, to 3.7% in 2017, and to 1.9% in 2019 (Table 2). Similarly, there was a steady yearly geometric increase in the number of LC from seven in 2016 to nine in 2017, 12 in 2018, and peaking at 18 in 2019, giving an increase of more than 250%. Out of the total 96 cholecystectomies in 20 years, we observed an increase in the trend, with LC accounting for 7.29% in 2016 (the first year of its deployment) to 9.38% in 2017, 12.5% in 2018, and 18.75% in 2019 (Table 1, Figure 1). The introduction of laparoscopy has led to a surge in the total number of cholecystectomies, more patients’ acceptance, and a rise in LC within four years. While there was a steady decline in the yearly rate of OC from three (30%) in 2016 to one (5.3%) in 2019, there was a marked increase in the yearly rate of LC from seven (70%) to 18 (94.7%) within the same period (Table 2).

This steady yearly rate of decline in OC from three (30%) to one (5.26%) observed in our study is comparable to that reported by Adisa et al. [7] in Ile-Ife, southwest Nigeria. Within a four-year period (2016-2019) when laparoscopic surgery was deployed in our center, 46 LCs were done out of a total of 54 cholecystectomies, accounting for 85.19% of all cholecystectomies (Table 1, Figure 2). These 46 LCs done within four years in our secondary healthcare facility was more than the two LCs in five years reported by Ngim et al. [8] in Calabar, Nigeria, and nine cholecystectomies done in three years by Ekwunife et al. [9] in Owerri, Nigeria, which are both tertiary healthcare institutions. Our result is, however, comparable to the 35 cases done in three years by Misauno [10] in a private hospital in Jos, in the same North Central region of Nigeria. The lack of awareness and exorbitant costs may be responsible for this regional disparity. Similarly, we found a rising trend in LC from seven (70%) to 18 (94.74%) of all cholecystectomies in four years (2016-2019), higher than that recorded by Adisa et al. [7] in Ile-Ife, Nigeria, who found a progressive increase in the number and rate of LC from 0% to 90% in 10 years. Perhaps the uptake and rate of LC in a suburban town like Ile-Ife, Nigeria are lower compared to that from Asokoro in Abuja, Nigeria, which is a high urban metropolis and the nation’s capital city probably because of the ease of accessibility of Abuja, subsidy, and the reduction in the cost of LC in Asokoro District Hospital.

Whereas the cost of OC in Asokoro District Hospital, Abuja is covered by both the National Health Insurance Scheme (NHIS) and FCT Health Insurance Scheme (FHIS), all patients accessing laparoscopic services in our center pay out-of-their-pockets as neither the NHIS nor the FHIS cover LC. However, this out-of-pocket payment did not reduce the trend of LC as one would have expected. The overall increase in cholecystectomy rate, with the reduced cost of LC in our facility, may be because patients now have access to LC at a lower cost compared to big private hospitals providing such services.

The impact of reducing the cost of LC for our patients during the period under review may have also positively influenced the remarkable uptake in our study. In addition to this, the advantages of a shorter hospital stay of two to three days, acceptable cosmetic scars, economic benefits to the patients, as well as fewer morbidities such as reduced postoperative pain and surgical site infections may have influenced the choice of more LCs. Other reports from Nigeria, the West African sub-region, and international studies have shown various increased trends in LCs because of the advantages of health insurance coverage and being a minimally invasive procedure with the advantages stated earlier. These factors undoubtedly facilitated an earlier return to work and increased patients’ demand for LC [11-15].

Although Lam et al. [13] in Scotland, UK reported an increase not only in the total cholecystectomies but also that of OC and LC, our study shows that LC and total cholecystectomy were on a steady increase with OC on the decline. This suggests that our patients preferred LC which may also be the reason for the rise in total cholecystectomies. The rising trend in Scotland, according to Lam et al., is attributed to an enhanced cost efficacy of LC, reduced surgical threshold, and that surgeons may be treating asymptomatic gallstone disease or resorting to LC as a diagnostic therapeutic test. The authors further stated that it is difficult to accept this explanation from Scotland without any material evidence that patients with asymptomatic gallstone diseases are being subjected to LC. Other possible explanations according to the study include increased referral by general practitioners and gastroenterologists resulting from enhanced perceived benefits of LC. This may also lead to diminished recourse to alternative non-surgical therapy (extracorporeal lithotripsy and oral bile salt therapy) and increased patient acceptability so that few patients refuse surgical treatment [13]. Similarly, Al-Mulhim et al. [14] in Saudi Arabia reported an increase in the rate of cholecystectomy in all age groups and both sexes after the introduction of LC.

The higher rate of LC in our study was influenced by the reduction in the cost of laparoscopic surgery in addition to other advantages such as reduced postoperative pain, acceptable cosmetic scars, shorter hospital stay, fewer morbidities, and higher economic benefits to the patients and society.

The limitations of this study are the small sample size, the short duration of the laparoscopic service, and being a retrospective study. Future studies are necessary to correlate these factors with the present trend.

## Conclusions

Appropriate unit costing by subsidizing LC and improved outcomes have led to a steady drop in OC and a rise in LCs and the total number of cholecystectomies in our center. The acquisition of the capacity for infrastructure and staffing for LC in other secondary healthcare facilities will further increase access in Nigeria and in the sub-region.
